# Psychosocial factors affecting sleep misperception in middle-aged community-dwelling adults

**DOI:** 10.1371/journal.pone.0241237

**Published:** 2020-10-23

**Authors:** Sungjong Park, Kyungmee Park, Jee-Seon Shim, Yoosik Youm, Junsol Kim, Eun Lee, Hyeon Chang Kim

**Affiliations:** 1 Department of Psychiatry and Institute of Behavioral Science in Medicine, Severance Hospital, Yonsei University College of Medicine, Seoul, Korea; 2 Department of Preventive Medicine, Yonsei University College of Medicine, Seoul, Korea; 3 Cardiovascular and Metabolic Diseases Etiology Research Center, Yonsei University College of Medicine, Seoul, Korea; 4 Department of Sociology, Yonsei University College of Social Sciences, Seoul, Korea; University of Sao Paulo Medical School, BRAZIL

## Abstract

Sleep misperception has long been a major issue in the field of insomnia research. Most studies of sleep misperception examine sleep underestimation by comparing the results of polysomnography conducted in a laboratory environment with patients’ sleep diary entries. We aimed to investigate psychosocial characteristics of adults who underestimated or overestimated sleep time in a nonclinical, middle-aged community-dwelling population. We collected one week of sleep data with wrist-worn accelerometers. We used egocentric social network analysis to analyze the effects of psychosocial factors. Among 4,060 study participants, 922 completed the accelerometer substudy. Underestimation was defined as an accelerometer-measured sleep time ≥ 6 h and a subjective sleep time < 6 h. Overestimation was defined as an objective sleep time < 6 h and a subjective sleep time ≥ 6 h. Psychosocial characteristics of the sleep misperception group were evaluated using multivariate regression analysis. A total of 47 participants underestimated sleep time, and 420 overestimated sleep time. Regression analysis revealed that women, living with spouse, economic satisfaction, and bridging potential had protective effects against sleep underestimation. Blame from a spouse involved a 3.8-times higher risk of underestimation than the control group (p = 0.002). In men, discussing concerns with a spouse had a protective effect against underestimation (p < 0.001). Economic satisfaction, feeling social network-based intimacy, and support from a spouse were associated with overestimation in women. In men, feeling social network-based intimacy was also associated with overestimation (p < 0.001). We found that social relationship quality was related to sleep overestimation and underestimation. This association was marked in women. Good social relationships may have positive effects on sleep misperception via attenuation of negative emotional reactions and effects on emotional regulation.

## Introduction

Insomnia disorder is a common mental health problem with a worldwide prevalence of 20% to 40%. This condition can co-occur with psychiatric disorders (e.g., depression, cognitive dysfunction, and impairment of attention and executive function) and medical disorders (e.g., cardiovascular disease) [1–5]. Insomnia has been defined as subjective discomfort associated with sleep initiation or maintenance experienced by an individual. Objective measures of sleep like polysomnography (PSG) or actigraphy are not always used in the evaluation of insomnia because insomnia disorder diagnosis and treatment outcomes are determined using subjective measures. Use of objective measures of a disease is always important. However, the subject of whether objective or subjective measures are more important in the study of insomnia remains controversial [6].

Results using subjective sleep quality or quantity measures do not always coincide with those of objectively measured sleep [7–9]. Sleep misperception is the discrepancy between the estimates of time for subjectively reported sleep and objectively measured sleep. In patients with insomnia, it usually presents as an underestimation of total sleep time or overestimation of wake time after sleep onset or sleep onset latency [10]. Sleep misperception is a very common phenomenon that can occur in >25% of primary insomnia patients [11,12]. Sleep misperception is important because it has a high prevalence and may have a critical role in the progression of insomnia [13]. Harvey et al. suggested that the tendency to misperceive sleep time causes an individual to mistakenly believe they did not have sufficient sleep the previous night [13]. It increases worry and anxiety about sleep quality. The resulting state of anxiety is associated with too much attention paid to factors that reduce sleep time. The excessive increased anxiety and worry worsens insomnia by facilitating detection of meaningless sensations or cues [14]. Given that this cycle of misperceived sleep time and increased anxiety can perpetuate insomnia, it is essential to understand and respond to characteristics of sleep misperception to improve insomnia treatment.

There are no clearly established theories about the mechanisms of sleep misperception. However, previous studies found several factors related to sleep misperception in patients with insomnia. Most are internal factors (e.g., psychiatric/medical comorbidities or subclinical psychological problems) that can cause mismatches between perceived and measured sleep times [15,16]. Insomnia is also affected by social factors, such as marital status, social support, and social connectedness [17–19]. Unfavorable social factors can act as psychological stressors and cause insomnia, regardless of whether an individual has depression or anxiety [20]. Studies of sleep misperception have not included these social factors [20,21].

Most sleep misperception studies have only included patients with insomnia who underestimate their sleep time [7,12,22,23]. In addition, the objective criteria for sleep in all the studies were made with PSG, which has limitations in terms of accessibility and familiarity. This limitation is partially attributable to the use of objective criteria and PSG in the experimental setting. Given that sleep misperception is a very common problem, it is likely to be prevalent in the general population as well as in patients with insomnia [24]. Sleep misperception can also result in sleep overestimation [25,26]. Investigation of sleep overestimation may be helpful to alleviate negative consequences associated with subjective sleep estimate errors.

Social network analysis can be used for objective and systematic identification of social factors. This structural analysis method focuses on relationship research and is used to derive characteristics of social networks by measuring information about individual relationships. Egocentric network analysis is a questionnaire-based method used to study these individual network characteristics. Social network size and bridging potential are the main variables examined using this analysis method. Social network size is the absolute number of people who most often discuss their important issues. Bridging potential is a tie between parties, which increases independence in everyday social life [27,28]. No studies have looked at the effects of social networks on sleep, but there are studies that have looked at the effects of social networks on severe mental illness [29,30].

We investigated social and psychological characteristics of people who underestimated or overestimated sleep in the Cardiovascular and Metabolic Diseases Etiology Research Center (CMERC) cohort. This cohort consisted of nonclinical, middle-aged community-dwelling individuals [31]. We collected one week of objectively measured sleep data using accelerometer instead of PSG. To investigate psychosocial factors, we used egocentric social network analysis to systematically assess individuals’ qualitative social characteristics (e.g., perceived social support and relationship problems) [32].

## Materials and methods

### Study population and design

The CMERC cohort was developed to identify novel risk factors and to develop evidence-based prevention strategies for cardiovascular and metabolic diseases in Korea. Data were collected from the cohort from July 2013 to June 2018 [31]. The cohort consisted of community-dwelling middle-aged adults excluding those with cardiovascular disease, a history of myocardial infarction, heart failure, stroke, or transient ischemic infarction, diagnosed with cancer in the previous 2 years, or who were currently being treated for any of the above conditions. Adults aged 30 to 64 years who resided at their current residence for at least 8 months, had no plans to move out of the study area in the next 2 years, and who could articulate their intention regarding study participation were eligible for the study. The study protocols were approved by the Institutional Review Boards of Severance Hospital, Yonsei University Health System, Seoul, Korea (4-2013-0661). Written informed consent was obtained from all participants before baseline measurements were taken. The baseline measures consisted of socio-demographic factors, medical history, health-related behaviors, psychological factors including depression (Beck Depression Inventory-II [BDI] score ≥ 14) [33] and quality of marital relationship, social network and support, anthropometry, and body composition. Results of cardiovascular examinations, blood analysis, and urinalysis were also included.

Among the 4,060 participants, 1,626 participated in a sub-study to check physical activity using wrist-worn accelerometers; 922 participants completed the study for 7 consecutive days. We mutually excluded individuals who self-reported histories of psychotropic medication prescription (antidepressants or sedative-hypnotics that could affect participant sleep, N = 22). We also excluded individuals with extremely low accelerometer-recorded sleeping times (<2 h total during the 7 days) because of possible errors in machine operation (N = 23) [11]. Among the excluded participants, 10 were in both groups. Data from 887 participants were included in the final analysis.

### Sleep characteristics

#### Self-reported sleep characteristics

For the cohort survey, all participants completed the self-reported questionnaires about sleep characteristics during the past year. Based on survey results, the average bed time and wake time, and total sleep time were collected for each participant. We defined reported sleep time as the subjective total sleep time. Respondents also answered questions about how difficult it was to fall asleep and to maintain sleep during the last week. The respondents chose one of four answers: never, sometimes (1−2 days per week), often (3−4 days per week), and almost every day (≥5 days per week). For the analysis, we dichotomized the categories into ‘yes’ (often or almost every day) and ‘no’ (never or sometimes). To screen for obstructive sleep apnea, questions about participants’ snoring patterns, daytime fatigue, and sleepiness while driving, which were based on the Berlin apnea questionnaire, were also included [34].

#### Accelerometer-measured sleep characteristics

Each CMERC participant wore a GENEActiv accelerometer (Activinsights Ltd., Kimbolton, UK) on their nondominant wrist day and night for 7 consecutive days. Use of an accelerometer is a valid, cost-effective, and convenient method. Accelerometer-measured sleep parameter results highly correspond to results using PSG. In previous studies, sleep parameters, such as sleep onset time and sleep duration, measured using GENEActiv were not significantly different from those measured using PSG [35,36]. The GENEActiv triaxial accelerometer recorded movement in three mutual vertical axes, environmental temperature, and light exposure, and was programmed to collect data at a frequency of 100 Hz. The raw accelerometer data were downloaded and scored in 1-min epoch files via post-processing software developed by the accelerometer manufacturer [37]. Time-to-bed, wake-up time, and total sleep time were determined based on metabolic equivalents task units, which represented the energy costs of an individual’s physical activities. Signal vector magnitude measurement was used to indicate behavioral changes [38]. In our study, we focused on total sleep time and total bed time, which have most often been used in previous studies. We calculated sleep efficiency by dividing total sleep time by total bed time. To obtain measured total sleep time and sleep efficiency, we calculated the average of 7 days of the measured values and defined the results as objective total sleep time and objective sleep efficiency.

#### Definitions of sleep underestimation and overestimation

Previous research on sleep studies has determined the standard value of sufficient sleep. Most studies have defined 6–7 hours as the minimum time for sufficient sleep. Focusing on previous studies, we defined the standard time of sleep misperception based on 6 hours of objective and subjective sleep [39–41]. Among those with an objectively measured total sleep time ≥6 h, we divided participants into one of two groups: subjective total sleep time <6 h or ≥6 h. We defined the former group as the sleep underestimation group and the latter as a control group. We also divided the group of participants with objectively measured total sleep time <6 h into one of two groups: subjective total sleep time ≥6 h or <6 h. The former group was defined as the sleep overestimation group and the latter was a control group.

### Psychosocial factors

Due to the nature of the cohort, which consisted mostly of middle-aged people, social factors included marriage-related questions (e.g., marital status, relationship with spouse). All participants answered the questions about social relationships, including the egocentric social network and marriage variables. The egocentric social network analysis is a methodological approach used to understand the social structure, function, and composition of network ties around an individual [42]. The participants were all asked to identify up to five non-spousal network members with whom they had most often discussed their important issues. The social network size was calculated as the sum of the spouse (0 or 1) and the number of non-spousal members (0 to 5). They were also asked how close they felt to each of the members (up to six) in their network. The four categories were ‘not close’, ‘somewhat close’, ‘close’, and ‘very close’. We calculated the average intimacy score and used it in the analysis as a proxy for relationship satisfaction. We also assessed the value of bridging potential, which referred to the extent to which an individual was associated with people who were not directly connected to each other. We defined that a participant could act as a bridge in a network when he or she was connected to at least two individuals who otherwise were not connected to each other [43].

Marriage is a permanent relationship among two adults made by a social contract. It has a variety of psychological effects on an individual’s mental health [44,45]. Some studies found that for both sexes, married adults with more relationship problems tend to have more trouble with sleep [46]. Others found that a stable relationship status with a spouse independently correlates with better sleep quality and continuity in women [47]. We wanted to identify which of the marriage-related factors had different effects on sleep perception in men and women. In the cohort survey, the questionnaire about marital relationships included leisure time (how often do you spend leisure time with your spouse?), worry (how often do you discuss your concerns with your spouse?), support (how often do you depend on your spouse in difficult situations?), and blame (how often does your spouse blame you?). All these questions had four choices (i.e., ‘often’, ‘sometimes’, ‘not very often’, and ‘not at all’). We dichotomized the answers into the categories ‘yes’ (often, sometimes) and ‘no’ (not very often, not at all).

### Statistical analyses

Because the effects of psychosocial factors on sleep differ by sex, we first compared the baseline characteristics of participants based on sex. As continuous variables did not follow a normal distribution, the Mann-Whitney test and χ^2^-tests were used to compare continuous and categorical variables, respectively, between the two sexes [48]. The analyses to identify factors associated with sleep overestimation and underestimation were also performed separately, depending on the sex of the participants. After a univariate regression analysis of each psychosocial variable, we performed a multivariate regression analysis. In addition to the psychosocial factors related to our main interests (i.e., social network and marriage-related variables), age, educational status (high school or less), current smoking or drinking use (yes or no), obesity (body mass index ≥ 25 or less) [49], depression (Beck depression inventory-II score ≥ 14) [33], and self-reported sleep disturbances including high risk of obstructive sleep apnea measured using the Berlin questionnaire and difficulty in sleep induction and maintenance were also included the analysis as covariates. These variables were found to affect sleep misperception or thought by the study investigators and to be potentially significant independent variables [23]. All independent variables were imported into the model and significantly associated factors were identified using backward elimination procedures in multivariate analyses. The maximum variance inflation factor value was <2.5, so multicollinearity was not problematic. The data analyses were performed using SPSS version 25.0 software (SPSS Inc., Chicago, IL), and the threshold for statistical significance was set at p < 0.05.

## Results

The results for the characteristics included in the final analysis are presented in Table 1. Sleep-related characteristics and psychosocial factors, including social network size and marital relationship, were significantly different between the two sexes. From a total of 887 participants included in the analyses, 47 underestimated their sleep time. Thirty-four participants in the underestimation group were women, which was 5.8% of all women. Thirteen male participants (4.3%) underestimated sleep time. This result was not significantly different from the rate of underestimation for women (p = 0.367). A total of 420 participants overestimated sleep time; 247 of them were women (42%) and 173 were men (58%). This sex-based difference was statistically significant (p < 0.001). The results for the characteristics of those who underestimated and overestimated sleep time are presented in S1 and S2 Tables.

**Table 1:** Characteristics of participants of each sex.

		Women (n = 588)	Men (n = 299)	*P* ^a^
Demographic factors	Age (years)	53.38 ± 8.41	52.55 ± 9.72	0.804
	Marital status, living with spouse	491 (83.50)	283 (94.65)	<0.001
	Education ≥ high school	506 (86.05)	284 (94.98)	<0.001
	Economic status, satisfactory	421 (71.60)	226 (75.59)	0.231
	BMI ≥ 25 (Kg/m^2^)	188 (31.97)	145 (48.49)	<0.001
	Smoking, current	10 (1.70)	70 (23.41)	<0.001
	Drinking, current	421 (71.60)	258 (86.29)	<0.001
Sleep-related factors	Total sleep time (mins)^b^	353.45 ± 87.52	330.54 ± 136.44	<0.001
	Sleep efficiency^b^	0.65 ± 0.14	0.58 ± 0.16	<0.001
	Self-reported total sleep time (mins)	404.93 ± 67.23	403.55 ± 64.38	0.779
	Berlin score, high risk^c^	94 (15.99)	88 (29.43)	<0.001
	Difficulty in sleep induction^d^	99 (16.84)	27 (9.03)	0.002
	Difficulty in sleep maintenance^d^	82 (13.96)	22 (7.36)	0.004
Psychosocial factors	BDI ≥ 14	175 (29.76)	47 (15.77)	<0.001
	Social network size	4.07 ± 1.55	3.80 ± 1.70	0.01
	Feeling intimacy in social network^e^	3.24 ± 0.65	3.28 ± 0.64	0.451
	Bridging potential, yes	407 (69.22)	206 (68.90)	0.922
	Having friends (≥1) outside of family	412 (70.07)	189 (63.21)	0.039
	Sharing leisure time with spouse	365 (62.07)	232 (77.59)	<0.001
	Discussing concerns with spouse	459 (78.06)	269 (89.97)	<0.001
	Support from spouse	441 (75.00)	248 (82.94)	0.007
	Blame from spouse	145 (24.66)	105 (35.12)	0.001

Abbreviations: BMI, body mass index; BDI, Beck Depression Inventory; mins, minutes.

Values are expressed as mean ± standard deviation for continuous variables or as n (%) for categorical variables.

^a^ p values were calculated with Mann-Whitney tests for continuous variables and χ^2^-tests for categorical variables.

^b^ measured by accelerometer.

^c^ 2 or more of the categories are positive.

^d^ 3 days or more per week.

^e^ range 1–4 points.

The results for the factors that were significantly related to sleep underestimation are presented in Table 2. The complete results for the univariate and multivariate analyses are presented in S3 and S4 Tables. Psychosocial factors had significant effects on sleep underestimation, especially in women. Women who lived with a spouse were 78% less likely to underestimate sleep time. Satisfaction with household economic status was negatively related to sleep underestimation in women. Women who reported blame by their spouses were 3.84 times more likely to underestimate sleep time than women who did not. Women with bridging potential had a 65% lower chance of sleep underestimation than women with no bridging potential. Men were less likely to underestimate sleep time when they discussed their concerns with their spouses.

**Table 2:** Factors associated with total sleep time underestimation.

	Women		Men	
	Adjusted OR (95% CI)	*P*	Adjusted OR (95% CI)	*P*
Marital status, living with spouse	0.22 (0.08 to 0.59)	0.003		
Economic status, satisfactory	0.37 (0.18 to 0.76)	0.006		
BMI ≥ 25 kg/m^2^	0.36 (0.13 to 0.95)	0.039		
Berlin score, high risk	2.86 (0.98 to 8.33)	0.054		
Difficulty in sleep maintenance			17.1 (3.54 to 82.7)	<0.001
Discussing concerns with spouse			0.08 (0.03 to 0.19)	<0.001
Blame from spouse	3.84 (1.63 to 9.06)	0.002		
Bridging potential	0.35 (0.17 to 0.71)	0.004		

The table includes only statistically significant variables (full results are in the S3 and S4 Tables).

Abbreviations: BMI, body mass index; CI, Confidence Interval; OR, Odds ratio.

The results for the factors significantly associated with sleep overestimation are presented in Table 3. The effects of psychosocial factors on sleep overestimation were also marked in women than in men. Intimacy with social network members had a positive association with sleep overestimation in both sexes. Unlike men, women who got support from their spouses in difficult situations were more likely to overestimate sleep time. Satisfaction with household economic status was also positively associated with sleep overestimation in women, but not in men. The complete results for the univariate and multivariate analyses of sleep overestimation are presented in S5 and S6 Tables.

**Table 3:** Factors associated with total sleep time overestimation.

	Women		Men	
	Adjusted OR (95% CI)	*P*	Adjusted OR (95% CI)	*P*
Education ≥ high school	0.41 (0.17 to 0.98)	0.044		
Economic status, satisfactory	2.13 (1.12 to 4.05)	0.021		
BMI ≥ 25 kg/m^2^			0.43 (0.20 to 0.94)	0.033
Difficulty in sleep induction	0.24 (0.12 to 0.48)	<0.001		
Difficulty in sleep maintenance			0.30 (0.09 to 0.94)	0.04
Feeling intimacy in social network	1.52 (1.15 to 2.00)	0.003	1.93 (1.59 to 2.35)	<0.001
Support from spouse	2.07 (1.06 to 4.06)	0.034		

The table includes only statistically significant variables (full results are in the S5 and S6 Tables).

Abbreviations: CI, Confidence Interval; BMI, Body mass index; OR, Odds ratio.

## Discussion

In this study, we evaluated relationships between sleep misperception and psychosocial factors in a middle-aged nonclinical community-dwelling cohort. Good social relationships were associated with sleep underestimation and overestimation. The results were marked in women than in men.

Among the 364 participants who had an accelerometer-measured sleep time ≥6 h, 47 people underestimated sleep time. The subjectively reported sleep time was underestimated by an average of 206 min in men and 96 min in women, compared with the sleep time measured using accelerometer (S1 Table). Women, persons living with spouse, persons satisfied with economic status, and persons with bridging potential between unconnected people had protective effects against sleep underestimation. However, even when living with a spouse, blame from that spouse resulted in a four-fold increase in the risk of sleep underestimation. This result indicated that relationship quality had a greater effect on sleep underestimation than simply being in a relationship. In men, sharing worries with a spouse also had a protective effect on sleep underestimation. Previous study findings have revealed mechanisms associated with the effects of good social relationships on mental health [50]. First, social relationships affect network members by providing normative guidance on health-relevant behaviors, such as regular exercise. These social influences consequently have positive effects on mental health by exerting beneficial effects on individuals. Second, integration in a social network is associated with a positive psychological state, like a sense of belonging and recognition of self-worth. Third, social network participation modulates neuroendocrine responses to stressors, which in turn increases the capacity to protect oneself against distress. Considering that many life stressors are related to breaks in social relationships (e.g., divorce or the death of a loved one), emotional regulation is closely affected by social relationships as well as by an individual’s own ability. Close relationships with others can attenuate emotional distress through coregulation. During this process, close relationships help to maintain an optimal emotional state by regulating emotional arousal [51]. Harvey et al.’s findings suggest that emotional arousal level can affect sleep misperception regardless of an insomnia diagnosis [52]. Therefore, good social relationships could have a protective effect on sleep underestimation by downgrading excessive arousal, a key mechanism of chronic insomnia, through attenuating negative emotional reaction and helping emotional regulation.

Social network bridging potential is defined by the ability to act as a link between two people not connected to each other. Usually, individuals with bridging potential are believed to be independent, autonomous, and socially active [53]. Although bridging potential does not necessarily result in bridging behaviors, it is associated with good health (e.g., cognitive function and functional health), especially in older women [43]. Studies on older adults > 57 years of age found that women have more bridging potential than men, including more non-kin bridging potential [53]. Cornwell suggested that in older women, bridging potential boosts independence and control in daily social life. Our study of this middle-aged cohort did not find sex-based differences in bridging potential. Nevertheless, women have more non-family friends than men. Because this study did not perform a complete social network analysis and count the total number of bridging potential for each participant, it is possible that a sex-based difference in bridging potential was not revealed. No studies have revealed the mechanisms underlying bridging potential that can affect sleep misperception. Because greater bridging potential indicates more social activity, thereby reflecting more social activities during the daytime, it may attenuate the effects of negative factors on sleep underestimation by diverting excessive attention from negative emotions. Alternatively, through an association with psychological strength characteristics (e.g., autonomy, independence, and self-regulation), emotional arousal may be reduced via the effects of bridging potential. The effects of social position on emotional regulation, arousal, and sleep perception should be examined in future studies.

Of the 523 participants who had an accelerometer-measured sleep time < 6 h, 420 overestimated their sleep time. The subjectively reported sleep time overestimated actual sleep time by an average of 122 min in women and 146 min in men (S2 Table). Sleep overestimation was also affected by good social relationships. In men, social network-based intimacy increased the tendency to overestimate sleep by about 1.9 times. In women, social network-associated intimacy and support from a spouse were associated with an increased tendency to overestimate sleep (1.5 and 2.1 times, respectively). These results suggested that similar to the feeling of intimacy, support from a spouse in difficult situations was likely to serve as perceived social support. A sleep diary-based study of older adults found that perceived social support is associated with shorter sleep latency times, irrespective of an insomnia diagnosis [54]. Similar to the previously mentioned results for the underestimation of sleep time, good social relationships had a positive effect on sleep time perception; this effect was marked in women. Study results suggest that social relationships have greater effects on mental health in women than in men [50]. Compared with men, women are more likely to be affected by stressful negative social network-based components. Inversely, women are also more influenced by supportive social network-based components [54]. Women also tend to respond more sensitively to both positive and negative factors in a marriage than men [46,55]. Women with a highly critical spouse have a greater tendency to exhibit maladaptive coping behaviors and report poorer psychological adjustment [56]. Our findings also suggested that social relationships played a more important role in women than in men for both sleep underestimation and overestimation. Negative components of social relationships (e.g., criticism from a spouse) increased the risk of sleep underestimation; positive components (e.g., feeling intimacy with network members and support from a spouse) affected sleep time perception in a positive direction.

The multivariate regression model of psychosocial factors found that satisfaction with economic status was associated with reduced sleep underestimation and increased sleep overestimation in women. Economic status can also have gender-dependent effects in terms of the relationship between social relationships and mental health [57]. Women with poorer economic conditions are more likely to provide their resources to others in the network than to get help from the network. This behavior is more likely to harm than to help them.

This study had several limitations. First, because the CMERC cohort study was not designed primarily for sleep research, evaluation of sleep was limited. For indicators of objective sleep, we only obtained information about total sleep time, time in bed, and sleep efficiency. We could not obtain information on sleep onset latency or wake after sleep onset, which are also associated with sleep misperception [11]. For subjective sleep assessment, we only had information on participants’ average sleep time and the reported difficulties with sleep initiation and maintenance. Most importantly, our data could not confirm whether a participant was diagnosed with insomnia or not. Because of the lack of information on insomnia diagnosis, we were unable to determine the proportion of people diagnosed with insomnia among those with and without sleep misperception. If many insomnia patients were in the target population, it is possible that sleep overestimation occurred if time spent lying down while awake was mistaken for sleeping time [58]. Secondly, as the accelerometer study was conducted after the CMERC cohort survey, the assessment of subjective sleep patterns and the objective sleep assessments through the accelerometer were not performed at the same time. Further studies should be conducted on various measurements to evaluate subjective and objective sleep characteristics. Second, no information on sleep schedules during weekdays and weekends or on shift-work schedules was available. Third, because this study used cross-sectional data, it is not valid to use the results to predict causal relationships between variables. Fourth, other than marriage, no other information about family-based social relationships was available.

Nevertheless, to our knowledge, this is the first study to investigate relationships between psychosocial factors and sleep misperception, including both sleep underestimation and overestimation, in a nonclinical, middle-aged, community-dwelling population. Previous studies revealed characteristics of sleep misperception from the perspective of PSG findings [59–62]. Those studies were used to examine physiological aspects (changes in the frequencies of electroencephalogram patterns or in cyclic alternating patterns) of sleep misperception. Insomnia disorder is significantly affected by psychosocial factors. Therefore, as a type of insomnia disorder, sleep misperception can also be affected by psychosocial factors [63]. The positive effects of using a psychological approach to treat insomnia have been demonstrated in previous studies [64–66]. These study results are important because they suggest that psychological intervention can be used to modify sleep misperception. Modifying psychosocial factors may be a way to improve sleep underestimation. Sleep misperception is not limited to those with insomnia; it is a common phenomenon that can occur in the genera**l** population [24]. Considering that misperceived sleep escalates the worry of having insufficient sleep and consequently worsens sleep disturbance, interventions for sleep misperception should be initiated at the prodromal phase [67].

## Conclusion

In summary, we found that psychosocial factors were related to sleep misperception in both sexes in a nonclinical, middle-aged cohort study population. Good social relationships were associated with sleep underestimation and overestimation. The results were marked in women than in men. Studies that include various kinds of social interactions as potential covariates are needed to identify other psychosocial effects on sleep misperception.

## Supporting information

S1 Table Characteristics of the sleep underestimation group. (DOCX)

S2 Table Characteristics of the sleep overestimation group. (DOCX)

S3 Table Factors associated with total sleep time underestimation in women. (DOCX)

S4 Table Factors associated with total sleep time underestimation in men. (DOCX)

S5 Table Factors associated with total sleep time overestimation in women. (DOCX)

S6 Table Factors associated with total sleep time overestimation in men. (DOCX)

## References

[1] Chunhua X, Jiacui D, Xue L, Kai WJM. Impaired emotional memory and decision-making following primary insomnia. 2019;98. 10.1097/MD.0000000000016512 PMC6709269 31335727

[2] Cunnington D, Junge MF, Fernando ATJMJoA. Insomnia: prevalence, consequences and effective treatment. 2013;199: S36–S40. 10.5694/mja13.10718 24138364

[3] Li L, Wu C, Gan Y, Qu X, Lu ZJBp. Insomnia and the risk of depression: a meta-analysis of prospective cohort studies. 2016;16: 375. 10.1186/s12888-016-1075-3 PMC5097837 27816065

[4] Merrigan JM, Buysse DJ, Bird JC, Livingston EHJJ. Insomnia. 2013;309: 733–733. 10.1001/jama.2013.524 23423421

[5] Sarsour K, Morin CM, Foley K, Kalsekar A, Walsh JKJSm. Association of insomnia severity and comorbid medical and psychiatric disorders in a health plan-based sample: Insomnia severity and comorbidities. 2010;11: 69–74. 10.1016/j.sleep.2009.02.008 19410512

[6] Sateia MJ, Buysse DJ, Krystal AD, Neubauer DN, Heald JLJJoCSM. Clinical practice guideline for the pharmacologic treatment of chronic insomnia in adults: an American Academy of Sleep Medicine clinical practice guideline. 2017;13: 307–349. 10.5664/jcsm.6470 27998379 PMC5263087

[7] Edinger JD, Krystal AD. Subtyping primary insomnia: is sleep state misperception a distinct clinical entity? Sleep Med Rev. 2003;7: 203–214. 10.1053/smrv.2002.0253 . 12927120

[8] Frankel BL, Coursey RD, Buchbinder R, Snyder FJAoGP. Recorded and reported sleep in chronic primary insomnia. 1976;33: 615–623. 10.1001/archpsyc.1976.01770050067011 178287

[9] Carskadon MA, Dement WC, Mitler M, Guilleminault C, Zarcone VP, Spiegel RJAJP. Self-reports versus sleep laboratory findings in 122 drug-free subjects with complaints of chronic insomnia. 1976;133: 1382–1388. 10.1176/ajp.133.12.1382 185919

[10] Perlis M, Giles D, Mendelson W, Bootzin RR, Wyatt JJJosr. Psychophysiological insomnia: the behavioural model and a neurocognitive perspective. 1997;6: 179–188. 10.1046/j.1365-2869.1997.00045.x 9358396

[11] Moon H-J, Song ML, Cho YWJJoCN. Clinical characteristics of primary insomniacs with sleep-state misperception. 2015;11: 358–363. 10.3988/jcn.2015.11.4.358 26256663 PMC4596102

[12] Semler CN, Harvey AGJBr, therapy. Misperception of sleep can adversely affect daytime functioning in insomnia. 2005;43: 843–856. 10.1016/j.brat.2004.06.016 15896282

[13] Harvey AG. A cognitive model of insomnia. Behav Res Ther. 2002;40: 869–893. 10.1016/s0005-7967(01)00061-4 . 12186352

[14] Clark DM. Anxiety disorders: why they persist and how to treat them. Behav Res Ther. 1999;37 Suppl 1: S5–27. 10.1016/s0005-7967(99)00048-0 . 10402694

[15] Dorsey CM, Bootzin RRJBP. Subjective and psychophysiologic insomnia: an examination of sleep tendency and personality. 1997;41: 209–216. 10.1016/0006-3223(95)00659-1 9018392

[16] Harvey AG, Tang NKJPb. (Mis) perception of sleep in insomnia: A puzzle and a resolution. 2012;138: 77. 10.1037/a0025730 PMC3277880 21967449

[17] Dollander M. [Etiology of adult insomnia]. L'Encephale. 2002;28: 493–502 . 12506261

[18] Green MJ, Espie CA, Benzeval M. Social class and gender patterning of insomnia symptoms and psychiatric distress: a 20-year prospective cohort study. BMC Psychiatry. 2014;14: 152 10.1186/1471-244X-14-152 24884517 PMC4041899

[19] Hsieh Y-P, Lu W-H, Yen C-F. Psychosocial Determinants of Insomnia in Adolescents: Roles of Mental Health, Behavioral Health, and Social Environment. Frontiers in Neuroscience. 2019;13 10.3389/fnins.2019.00013 31447642 PMC6696979

[20] Bastien CH, Vallieres A, Morin CMJBsm. Precipitating factors of insomnia. 2004;2: 50–62. 10.1207/s15402010bsm0201_5 15600224

[21] Schirmer AJFiIN. How emotions change time. 2011;5: 58. 10.3389/fnint.2011.00058 PMC3207328 22065952

[22] Dittoni S, Mazza M, Losurdo A, Testani E, Di Giacopo R, Marano G, et al Psychological functioning measures in patients with primary insomnia and sleep state misperception. 2013;128: 54–60. 10.1111/ane.12078 23406317

[23] Fernandez-Mendoza J, Calhoun SL, Bixler EO, Karataraki M, Liao D, Vela-Bueno A, et al Sleep misperception and chronic insomnia in the general population: the role of objective sleep duration and psychological profiles. 2011;73: 88 10.1097/PSY.0b013e3181fe365a 20978224 PMC3408864

[24] Bianchi MT, Wang W, Klerman EBJJoCSM. Sleep misperception in healthy adults: implications for insomnia diagnosis. 2012;8: 547–554. 10.5664/jcsm.2154 23066367 PMC3459201

[25] Trajanovic NN, Radivojevic V, Kaushansky Y, Shapiro CMJSM. Positive sleep state misperception–a new concept of sleep misperception. 2007;8: 111–118 10.1016/j.sleep.2006.08.013 17275407

[26] Edinger JD, Fins AIJS. The distribution and clinical significance of sleep time misperceptions among insomniacs. 1995;18: 232–239. 10.1093/sleep/18.4.232 7618020

[27] Liu J. An introduction to school network analysis. Beijing: Social Science Documentation Publishing House. 2004.

[28] MA S-Q, JIAO C, ZHANG M-Q. Application of social network analysis in psychology. Advances in Psychological Science. 2011;19: 755–764.

[29] Sündermann O, Onwumere J, Kane F, Morgan C, Kuipers E. Social networks and support in first-episode psychosis: exploring the role of loneliness and anxiety. Social Psychiatry and Psychiatric Epidemiology. 2014;49: 359–366. 10.1007/s00127-013-0754-3 23955376 PMC4081600

[30] Lorant V, Nazroo J, Nicaise P, The Title107 Study G. Optimal Network for Patients with Severe Mental Illness: A Social Network Analysis. Administration and Policy in Mental Health and Mental Health Services Research. 2017;44: 877–887. 10.1007/s10488-017-0800-7 PMC5640746 28341927

[31] Shim JS, Song BM, Lee JH, Lee SW, Park JH, Choi DP, et al Cohort Profile: The Cardiovascular and Metabolic Diseases Etiology Research Center Cohort in Korea. Yonsei Med J. 2019;60: 804–810. 10.3349/ymj.2019.60.8.804 . 31347337 PMC6660443

[32] Schepis D. Social network analysis from a qualitative perspective. ANZMAC; 2011.

[33] Beck AT, Steer RA, Brown GKJSA. Beck depression inventory-II. 1996;78: 490–498.

[34] Netzer NC, Stoohs RA, Netzer CM, Clark K, Strohl KP. Using the Berlin Questionnaire To Identify Patients at Risk for the Sleep Apnea Syndrome. Annals of Internal Medicine. 1999;131: 485–491. 10.7326/0003-4819-131-7-199910050-00002 10507956

[35] van Hees VT, Sabia S, Jones SE, Wood AR, Anderson KN, Kivimäki M, et al Estimating sleep parameters using an accelerometer without sleep diary. Scientific reports. 2018;8: 1–11. 10.1038/s41598-017-17765-5 30154500 PMC6113241

[36] Van Hees VT, Sabia S, Anderson KN, Denton SJ, Oliver J, Catt M, et al A novel, open access method to assess sleep duration using a wrist-worn accelerometer. PloS one. 2015;10: e0142533 10.1371/journal.pone.0142533 26569414 PMC4646630

[37] Rosenberger ME, Buman MP, Haskell WL, McConnell MV, Carstensen LL. 24 hours of sleep, sedentary behavior, and physical activity with nine wearable devices. Medicine and science in sports and exercise. 2016;48: 457 10.1249/MSS.0000000000000778 26484953 PMC4760880

[38] Esliger DW, Rowlands AV, Hurst TL, Catt M, Murray P, Eston RGJM, et al Validation of the GENEA Accelerometer. 2011;43: 1085–1093. 10.1249/MSS.0b013e31820513be 21088628

[39] Itani O, Jike M, Watanabe N, Kaneita Y. Short sleep duration and health outcomes: a systematic review, meta-analysis, and meta-regression. Sleep medicine. 2017;32: 246–256. 10.1016/j.sleep.2016.08.006 27743803

[40] Cappuccio FP, D'Elia L, Strazzullo P, Miller MA. Sleep duration and all-cause mortality: a systematic review and meta-analysis of prospective studies. Sleep. 2010;33: 585–592. 10.1093/sleep/33.5.585 20469800 PMC2864873

[41] Cappuccio FP, D'Elia L, Strazzullo P, Miller MA. Quantity and quality of sleep and incidence of type 2 diabetes: a systematic review and meta-analysis. Diabetes care. 2010;33: 414–420. 10.2337/dc09-1124 19910503 PMC2809295

[42] Djomba JK, Zaletel-Kragelj L. A methodological approach to the analysis of egocentric social networks in public health research: a practical example. Zdr Varst. 2016;55: 256–263. 10.1515/sjph-2016-0035 . 27703548 PMC5030837

[43] Cornwell B. Network Bridging Potential in Later Life:Life-Course Experiences and Social Network Position. 2009;21: 129–154. 10.1177/0898264308328649 . 19144972 PMC2742371

[44] Gove WR, Hughes M, Style CB. Does marriage have positive effects on the psychological well-being of the individual? Journal of health and social behavior. 1983: 122–131. 6886367

[45] Uecker JE. Marriage and mental health among young adults. J Health Soc Behav. 2012;53: 67–83. 10.1177/0022146511419206 . 22328171 PMC3390929

[46] Troxel WM, Robles TF, Hall M, Buysse DJ. Marital quality and the marital bed: Examining the covariation between relationship quality and sleep. Sleep medicine reviews. 2007;11: 389–404. 10.1016/j.smrv.2007.05.002 17854738 PMC2644899

[47] Troxel WM, Buysse DJ, Matthews KA, Kravitz HM, Bromberger JT, Sowers M, et al Marital/cohabitation status and history in relation to sleep in midlife women. Sleep. 2010;33: 973–981. 10.1093/sleep/33.7.973 20614858 PMC2894440

[48] Suh S, Cho N, Zhang J. Sex Differences in Insomnia: from Epidemiology and Etiology to Intervention. Curr Psychiatry Rep. 2018;20: 69 10.1007/s11920-018-0940-9 . 30094679

[49] Seo MH, Lee WY, Kim SS, Kang JH, Kang JH, Kim KK, et al Corrigendum: 2018 Korean Society for the Study of Obesity Guideline for the Management of Obesity in Korea. J Obes Metab Syndr. 2019;28: 143 10.7570/jomes.2019.28.2.143 . 31294348 PMC6604844

[50] Kawachi I, Berkman LF. Social ties and mental health. J Urban Health. 2001;78: 458–467. 10.1093/jurban/78.3.458 . 11564849 PMC3455910

[51] Butler EA, Randall AK. Emotional Coregulation in Close Relationships. Emotion Review. 2012;5: 202–210. 10.1177/1754073912451630

[52] Harvey AG, Tang NK, Browning L. Cognitive approaches to insomnia. Clin Psychol Rev. 2005;25: 593–611. 10.1016/j.cpr.2005.04.005 . 15979771

[53] Cornwell B. Independence through social networks: bridging potential among older women and men. J Gerontol B Psychol Sci Soc Sci. 2011;66: 782–794. 10.1093/geronb/gbr111 . 21983039 PMC3198249

[54] Troxel WM, Buysse DJ, Monk TH, Begley A, Hall M. Does social support differentially affect sleep in older adults with versus without insomnia? J Psychosom Res. 2010;69: 459–466. 10.1016/j.jpsychores.2010.04.003 . 20955865 PMC2958100

[55] Kiecolt-Glaser JK, Newton TLJPb. Marriage and health: his and hers. 2001;127: 472 10.1037/0033-2909.127.4.472 11439708

[56] Manne SL, Zautra AJJJop, psychology s. Spouse criticism and support: their association with coping and psychological adjustment among women with rheumatoid arthritis. 1989;56: 608 10.1037//0022-3514.56.4.608 2709309

[57] Belle D. Gender differences in the social moderators of stress. In: Barnett RC BL, Baruch GK, editor. Gender and Stress. New York: The Free Press; 1987 pp. 257–277.

[58] Sadeh A. The role and validity of actigraphy in sleep medicine: an update. Sleep medicine reviews. 2011;15: 259–267. 10.1016/j.smrv.2010.10.001 21237680

[59] Bastien CH, Ceklic T, St-Hilaire P, Desmarais F, Pérusse AD, Lefrançois J, et al Insomnia and sleep misperception. Pathologie Biologie. 2014;62: 241–251. 10.1016/j.patbio.2014.07.003 25179115

[60] Feige B, Al-Shajlawi A, Nissen C, Voderholzer U, Hornyak M, Spiegelhalder K, et al Does REM sleep contribute to subjective wake time in primary insomnia? A comparison of polysomnographic and subjective sleep in 100 patients. Journal of Sleep Research. 2008;17: 180–190. 10.1111/j.1365-2869.2008.00651.x 18482106

[61] Krystal AD, Edinger JD, Wohlgemuth WK, Marsh GRJS. NREM sleep EEG frequency spectral correlates of sleep complaints in primary insomnia subtypes. 2002;25: 626–636. 12224842

[62] Tang NK, Harvey AG. Effects of cognitive arousal and physiological arousal on sleep perception. Sleep. 2004;27: 69–78. 10.1093/sleep/27.1.69 14998240

[63] Käppler C, Hohagen FJEaop, neuroscience c. Psychosocial aspects of insomnia. 2003;253: 49–52. 10.1007/s00406-003-0406-9 12664315

[64] Morgan K, Dixon S, Mathers N, Thompson J, Tomeny MJBJGP. Psychological treatment for insomnia in the management of long-term hypnotic drug use: a pragmatic randomised controlled trial. 2003;53: 923–928. 14960215 PMC1314744

[65] Espie CA, Brooks DN, Lindsay WRJJobt, psychiatry e. An evaluation of tailored psychological treatment of insomnia. 1989;20: 143–153. 10.1016/0005-7916(89)90047-5 2685045

[66] Morin CM, Bootzin RR, Buysse DJ, Edinger JD, Espie CA, Lichstein KLJS. Psychological and behavioral treatment of insomnia: update of the recent evidence (1998–2004). 2006;29: 1398–1414. 10.1093/sleep/29.11.1398 17162986

[67] Salin-Pascual RJ, Roehrs TA, Merlotti LA, Zorick F, Roth T. Long-term study of the sleep of insomnia patients with sleep state misperception and other insomnia patients. Am J Psychiatry. 1992;149: 904–908. 10.1176/ajp.149.7.904 . 1609869

