# Supplementary material for: Psychosocial factors affecting sleep misperception in middle-aged community-dwelling adults
Source: PLoS One. 2020 Oct 23;15(10):e0241237. doi: 10.1371/journal.pone.0241237 (PMC7584196; doi:10.1371/journal.pone.0241237)
Supplement: S1 Table — (DOCX) [file pone.0241237.s001.docx]

| **Supporting information**  **Supplement 1 Table.**  Characteristics of the sleep underestimation group. | | | | | | | |
| --- | --- | --- | --- | --- | --- | --- | --- |
|  | | Women | | | Men | | |
|  |  | underestimating group  (n = 34) | Control  (n = 241) | *P* ^a^ | underestimating group  (n = 13) | Control  (n = 76) | *P* ^a^ |
| Demographic factors | Age (years) | 54.59 ± 7.74 | 52.17 ± 9.29 | 0.180 | 54.62 ± 7.33 | 49.08 ± 10.20 | 0.087 |
|  | Marital status, living with spouse | 26 (81.25) | 190 (84.82) | 0.603 | 12 (92.31) | 73 (96.05) | 0.943 |
|  | Education ≥ high school | 30 (88.24) | 210 (87.14) | 0.857 | 11 (84.62) | 72 (94.74) | 0.179 |
|  | Economic status, satisfactory | 19 (55.88) | 173 (71.78) | 0.059 | 9 (69.23) | 62 (81.58) | 0.306 |
|  | BMI ≥ 25 (Kg/m^2^) | 8 (23.53) | 74 (30.71) | 0.392 | 4 (30.77) | 35 (46.05) | 0.305 |
|  | Smoking, current, yes | 0 (0.00) | 4 (1.66) | 0.449 | 3 (23.08) | 20 (26.32) | 0.805 |
|  | Drinking, current, yes | 21 (61.76) | 175 (72.61) | 0.191 | 10 (76.92) | 66 (86.84) | 0.349 |
| Sleep-related factors | Total sleep time (mins)^b^ | 401.61 ± 35.70 | 419.96 ± 78.31 | 0.095 | 506.38 ± 235.14 | 446.57 ± 174.85 | 0.236 |
|  | Sleep efficiency^b^ | 68.25 ± 14.11 | 71.77 ± 10.92 | 0.254 | 65.14 ± 14.78 | 68.75 ± 12.09 | 0.339 |
|  | Self-reported total sleep time (mins) | 305.44 ± 29.03 | 432.53 ± 48.05 | <0.001 | 300.00 ± 24.50 | 426.78 ± 55.80 | <0.001 |
|  | Berlin score, high risk^c^ | 7 (20.59) | 29 (12.03) | 0.166 | 3 (23.08) | 19 (25.00) | 0.882 |
|  | Difficulty in sleep induction^d^ | 11 (32.35) | 39 (16.18) | 0.022 | 4 (30.77) | 8 (10.53) | 0.048 |
|  | Difficulty in sleep maintenance^d^ | 11 (32.35) | 31 (12.86) | 0.003 | 6 (46.15) | 4 (5.26) | <0.001 |
| Psychosocial factors | BDI ≥ 14 | 9 (26.47) | 73 (30.29) | 0.649 | 3 (23.08) | 9 (11.84) | 0.273 |
|  | Social network size | 4.15 ± 1.54 | 3.96 ± 1.50 | 0.462 | 3.77 ± 1.74 | 3.87 ± 1.73 | 0.859 |
|  | Feeling intimacy in social network | 3.18 ± 0.52 | 3.32 ± 0.64 | 0.141 | 3.62 ± 0.51 | 3.29 ± 0.65 | 0.096 |
|  | Bridging potential, yes | 20 (58.82) | 169 (70.12) | 0.183 | 8 (61.54) | 51 (67.11) | 0.695 |
|  | Having friends (≥1) outside of family | 23 (67.65) | 161 (66.80) | 0.922 | 7 (53.85) | 43 (56.58) | 0.854 |
|  | Sharing leisure time with spouse | 27 (79.42) | 176 (73.03) | 0.428 | 10 (76.92) | 70 (92.11) | 0.093 |
|  | Discussing concerns with spouse | 23 (67.65) | 169 (70.12) | 0.768 | 9 (69.23) | 61 (80.26) | 0.370 |
|  | Support from spouse | 10 (29.42) | 56 (23.24) | 0.430 | 3 (23.08) | 24 (31.58) | 0.538 |
|  | Blame from spouse | 29 (85.29) | 214 (88.80) | 0.551 | 10 (76.92) | 69 (90.79) | 0.143 |
| Abbreviations: BMI, body mass index; BDI, Beck Depression Inventory; mins, minutes  Values are expressed as mean ± standard deviation for continuous variables and as n (%) for categorical variables.  ^a^ P values were calculated from independent-sample t-tests, Mann-Whitney tests for continuous variables or χ^2^-tests for categorical variables between underestimating group and controls for each sex.  ^b^ measured by accelerometer  ^c^ 2 or more of the categories are positive  ^d^ 3 days or more per week | | | | | | | |
