# Supplementary material for: Psychosocial factors affecting sleep misperception in middle-aged community-dwelling adults
Source: PLoS One. 2020 Oct 23;15(10):e0241237. doi: 10.1371/journal.pone.0241237 (PMC7584196; doi:10.1371/journal.pone.0241237)
Supplement: S2 Table — (DOCX) [file pone.0241237.s002.docx]

| **Supplement 2 Table.**  Characteristics of the sleep overestimation group. | | | | | | | |
| --- | --- | --- | --- | --- | --- | --- | --- |
|  | | Women | | | Men | | |
|  |  | overestimating group (n = 247) | Control  (n = 66) | *P* ^a^ | overestimating group  (n = 173) | Control  (n = 37) | *P* ^a^ |
| Demographic factors | Age (years) | 54.30 ± 7.83 | 53.76 ± 6.89 | 0.225 | 53.45 ± 9.85 | 54.70 ± 6.95 | 0.809 |
|  | Marital status, living with spouse | 222 (92.89) | 53 (82.81) | 0.013 | 164 (98.80) | 34 (97.14) | 0.464 |
|  | Education ≥ high school | 206 (83.40) | 60 (90.91) | 0.129 | 164 (94.80) | 37 (100.00) | 0.156 |
|  | Economic status, satisfactory | 189 (76.52) | 40 (60.61) | 0.010 | 129 (74.57) | 26 (70.27) | 0.590 |
|  | BMI ≥ 25 (Kg/m^2^) | 80 (32.39) | 26 (39.39) | 0.285 | 80 (46.24) | 26 (70.27) | 0.008 |
|  | Smoking, current, yes | 5 (2.02) | 1 (1.52) | 0.789 | 40 (23.12) | 7 (18.92) | 0.578 |
|  | Drinking, current, yes | 175 (70.85) | 50 (75.76) | 0.431 | 151 (87.28) | 31 (83.78) | 0.570 |
| Sleep-related factors | Total sleep time (mins) | 298.12 ± 51.54 | 292.87 ± 53.54 | 0.480 | 276.51 ± 53.47 | 283.01 ± 48.71 | 0.567 |
|  | Sleep efficiency | 59.91 ± 14.49 | 59.37 ± 15.81 | 0.808 | 52.62 ± 14.35 | 60.05 ± 15.63 | 0.005 |
|  | Self-reported total sleep time (mins) | 420.32 ± 52.21 | 297.80 ± 31.48 | <0.001 | 422.69 ± 44.36 | 302.70 ± 31.48 | <0.001 |
|  | Berlin score, high risk^c^ | 42 (17.00) | 16 (24.24) | 0.179 | 52 (30.06) | 14 (37.84) | 0.355 |
|  | Difficulty in sleep induction^d^ | 26 (10.53) | 23 (34.85) | <0.001 | 9 (5.20) | 6 (16.22) | 0.018 |
|  | Difficulty in sleep maintenance^d^ | 25 (10.12) | 15 (22.73) | 0.006 | 9 (5.20) | 3 (8.11) | 0.489 |
| Psychosocial factors | BDI ≥ 14 | 66 (26.72) | 27 (40.91) | 0.025 | 29 (16.86) | 6 (16.21) | 0.924 |
|  | Social network size | 4.28 ± 1.58 | 4.00 ± 1.66 | 0.217 | 3.79 ± 1.67 | 3.70 ± 1.60 | 0.782 |
|  | Feeling intimacy in social network | 3.23 ± 0.65 | 3.05 ± 0.67 | 0.045 | 3.26 ± 0.64) | 3.22 ± 0.63 | 0.656 |
|  | Bridging potential, yes | 169 (68.42) | 49 (74.24) | 0.361 | 118 (68.21) | 29 (78.38) | 0.220 |
|  | Having friends (≥1) outside of family | 181 (73.28) | 47 (71.21) | 0.737 | 112 (64.74) | 27 (72.97) | 0.337 |
|  | Sharing leisure time with spouse | 208 (84.21) | 48 (72.73) | 0.032 | 155 (89.60) | 34 (91.89) | 0.673 |
|  | Discussing concerns with spouse | 205 (83.00) | 44 (66.67) | 0.003 | 148 (85.55) | 30 (81.08) | 0.492 |
|  | Support from spouse | 61 (24.70) | 22 (33.33) | 0.158 | 52 (30.06) | 12 (32.43) | 0.776 |
|  | Blame from spouse | 221 (89.47) | 60 (90.91) | 0.732 | 155 (89.60) | 33 (89.19) | 0.942 |
| Abbreviations: BMI, body mass index; BDI, Beck Depression Inventory; mins, minutes  Values are expressed as mean ± standard deviation for continuous variables and as n (%) for categorical variables.  ^a^ P values were calculated with independent-sample t-tests, Mann-Whitney tests for continuous variables or χ^2^-tests for categorical variables between underestimating group and controls in each sex.  ^b^ measured by accelerometer  ^c^ 2 or more of the categories are positive  ^d^ 3 days or more per week | | | | | | | |
