# Supplementary material for: Psychosocial factors affecting sleep misperception in middle-aged community-dwelling adults
Source: PLoS One. 2020 Oct 23;15(10):e0241237. doi: 10.1371/journal.pone.0241237 (PMC7584196; doi:10.1371/journal.pone.0241237)
Supplement: S3 Table — (DOCX) [file pone.0241237.s003.docx]

| **Supplement 3 Table.**  Factors associated with total sleep time underestimation in women. | | | | |
| --- | --- | --- | --- | --- |
|  | Univariate Model | | Multivariate Model | |
|  | OR (95% CI) | *P* | Adjusted OR (95% CI) | *P* |
| Age | 1.00 (0.96 to 1.04) | 0.888 | - | - |
| Marital status, living with spouse | 0.21 (0.44 to 0.97) | 0.045 | 0.22 (0.08 to 0.59) | 0.003 |
| Education ≥ high school | 1.43 (0.41 to 4.96) | 0.575 | - | - |
| Economic status, satisfactory | 0.42 (0.17 to 1.05) | 0.063 | 0.37 (0.18 to 0.76) | 0.006 |
| BMI ≥ 25 kg/m^2^ | 0.43 (0.15 to 1.20) | 0.106 | 0.36 (0.13 to 0.95) | 0.039 |
| Smoking, current | 0.00 | 0.999 | - | - |
| Drinking, current | 0.73 (0.31 to 1.72) | 0.472 | - | - |
| BDI ≥ 14 | 0.42 (0.15 to 1.17) | 0.097 | 0.49 (0.20 to 1.18) | 0.112 |
| Berlin score, high risk | 2.09 (0.62 to 7.08) | 0.237 | 2.86 (0.98 to 8.33) | 0.054 |
| Difficulty in sleep induction | 1.62 (0.50 to 5.31) | 0.424 | - | - |
| Difficulty in sleep maintenance | 1.71 (0.54 to 5.46) | 0.364 | - | - |
| Social network size | 1.11 (0.83 to 1.50) | 0.477 | - | - |
| Feeling intimacy in social network | 0.70 (0.38 to 1.29) | 0.256 | - | - |
| Sharing leisure time with spouse | 2.06 (0.72 to 5.88) | 0.179 | 2.18 (0.84 to 5.67) | 0.109 |
| Discussing concerns with spouse | 3.08 (0.56 to 16.89) | 0.196 | - | - |
| Support from spouse | 0.44 (0.12 to 1.58) | 0.210 | - | - |
| Blame from spouse | 3.59 (1.46 to 8.84) | 0.005 | 3.84 (1.63 to 9.06) | 0.002 |
| Having friends (≥1) outside of family | 1.40 (0.53 to 3.70) | 0.497 | - | - |
| Bridging potential, yes | 0.39 (0.15 to 0.97) | 0.043 | 0.35 (0.17 to 0.71) | 0.004 |
| Abbreviations: BMI, body mass index; BDI, Beck Depression Inventory; OR, Odds ratio; CI, Confidence Interval | | | | |
