# Supplementary material for: Psychosocial factors affecting sleep misperception in middle-aged community-dwelling adults
Source: PLoS One. 2020 Oct 23;15(10):e0241237. doi: 10.1371/journal.pone.0241237 (PMC7584196; doi:10.1371/journal.pone.0241237)
Supplement: S4 Table — (DOCX) [file pone.0241237.s004.docx]

| **Supplement 4 Table.**  Factors associated with total sleep time underestimation in men. | | | | |
| --- | --- | --- | --- | --- |
|  | Univariate Model | | Multivariate Model | |
|  | OR (95% CI) | *P* | Adjusted OR (95% CI) | *P* |
| Age | 1.05 (0.97 to 1.14) | 0.259 | - | - |
| Marital status, living with spouse |  |  | - | - |
| Education ≥ high school | 0.72 (0.00 to 1.38) | 0.080 | - | - |
| Economic status, satisfactory | 0.44 (0.07 to 2.88) | 0.393 | - | - |
| BMI ≥ 25 kg/m^2^ | 1.03 (0.20 to 5.44) | 0.973 | - | - |
| Smoking, current | 0.59 (0.05 to 6.91) | 0.675 | - | - |
| Drinking, current | 0.67 (0.07 to 6.44) | 0.730 | - | - |
| BDI ≥ 14 | 0.83 (0.08 to 8.29) | 0.872 | - | - |
| Berlin score, high risk | 0.75 (0.08 to 6.86) | 0.795 | - | - |
| Difficulty in sleep induction | 0.24 (0.01 to 4.05) | 0.323 | - | - |
| Difficulty in sleep maintenance | 60.91 (3.20 to 1157.99) | 0.006 | 17.12 (3.54 to 82.74) | <0.001 |
| Social network size | 0.87 (0.46 to 1.62) | 0.653 | - | - |
| Feeling intimacy in social network | 1.67 (0.56 to 4.93) | 0.355 | - | - |
| Sharing leisure time with spouse | 0.76 (0.11 to 4.72) | 0.718 | - | - |
| Discussing concerns with spouse | 0.06 (0.00 to 1.25) | 0.069 | 0.08 (0.03 to 0.19) | <0.001 |
| Support from spouse | 0.28 (0.03 to 2.76) | 0.276 | - | - |
| Blame from spouse | 1.35 (0.25 to 7.44) | 0.730 | - | - |
| Having friends (≥1) outside of family | 1.04 (0.13 to 8.51) | 0.968 | - | - |
| Bridging potential, yes | 4.66 (0.44 to 49.11) | 0.201 | - | - |
| Abbreviations: BMI, body mass index; BDI, Beck Depression Inventory; OR, Odds ratio; CI, Confidence Interval | | | | |
