# Supplementary material for: Psychosocial factors affecting sleep misperception in middle-aged community-dwelling adults
Source: PLoS One. 2020 Oct 23;15(10):e0241237. doi: 10.1371/journal.pone.0241237 (PMC7584196; doi:10.1371/journal.pone.0241237)
Supplement: S5 Table — (DOCX) [file pone.0241237.s005.docx]

| **Supplement 5 Table.**  Factors associated with total sleep time overestimation in women. | | | | |
| --- | --- | --- | --- | --- |
|  | Univariate Model | | Multivariate Model | |
|  | OR (95% CI) | *P* | Adjusted OR (95% CI) | *P* |
| Age | 1.01 (0.98 to 1.04) | 0.394 | - | - |
| Marital status, living with spouse | 1.02 (0.26 to 4.06) | 0.974 | - | - |
| Education ≥ high school | 0.39 (0.14 to 1.03) | 0.056 | 0.41 (0.17 to 0.98) | 0.044 |
| Economic status, satisfactory | 2.15 (1.09 to 4.25) | 0.028 | 2.13 (1.12 to 4.05) | 0.021 |
| BMI ≥ 25 kg/m^2^ | 0.67 (0.35 to 1.30) | 0.236 | - | - |
| Smoking, current | 0.87 (0.09 to 8.79) | 0.907 | - | - |
| Drinking, current | 0.81 (0.41 to 1.60) | 0.536 | - | - |
| BDI ≥ 14 | 0.71 (0.37 to 1.40) | 0.324 | - | - |
| Berlin score, high risk | 0.73 (0.34 to 1.55) | 0.408 | - | - |
| Difficulty in sleep induction | 0.27 (0.12 to 0.61) | 0.001 | 0.24 (0.12 to 0.48) | <0.001 |
| Difficulty in sleep maintenance | 0.66 (0.27 to 1.63) | 0.366 | - | - |
| Social network size | 1.00 (0.80 to 1.24) | 0.967 | - | - |
| Feeling intimacy in social network | 1.52 (1.00 to 2.32) | 0.050 | 1.52 (1.15 to 2.00) | 0.003 |
| Sharing leisure time with spouse | 0.84 (0.37 to 1.94) | 0.687 | - | - |
| Discussing concerns with spouse | 1.07 (0.31 to 3.70) | 0.917 | - | - |
| Support from spouse | 1.94 (0.64 to 5.85) | 0.241 | 2.07 (1.06 to 4.06) | 0.034 |
| Blame from spouse | 1.60 (0.73 to 3.50) | 0.244 | - | - |
| Having friends (≥1) outside of family | 0.99 (0.47 to 2.07) | 0.970 | - | - |
| Bridging potential, yes | 0.87 (0.42 to 1.80) | 0.703 | - | - |
| Abbreviations: BMI, body mass index; BDI, Beck Depression Inventory; OR, Odds ratio; CI, Confidence Interval | | | | |
