# Supplementary material for: Psychosocial factors affecting sleep misperception in middle-aged community-dwelling adults
Source: PLoS One. 2020 Oct 23;15(10):e0241237. doi: 10.1371/journal.pone.0241237 (PMC7584196; doi:10.1371/journal.pone.0241237)
Supplement: S6 Table — (DOCX) [file pone.0241237.s006.docx]

| **Supplement 6 Table.**  Factors associated with total sleep time overestimation in men. | | | | |
| --- | --- | --- | --- | --- |
|  | Univariate Model | | Multivariate Model | |
|  | OR (95% CI) | *P* | Adjusted OR (95% CI) | *P* |
| Age | 1.00 (0.97 to 1.05) | 0.829 | - | - |
| Marital status, living with spouse | 2.41 (0.17 to 34.36) | 0.517 | - | - |
| Education ≥ high school | 0.25 (0.02 to 3.30) | 0.292 | - | - |
| Economic status, satisfactory | 1.39 (0.51 to 3.75) | 0.521 | - | - |
| BMI ≥ 25 kg/m^2^ | 0.37 (0.16 to 0.90) | 0.029 | 0.43 (0.20 to 0.94) | 0.033 |
| Smoking, current | 2.18 (0.72 to 6.66) | 0.169 | - | - |
| Drinking, current | 2.15 (0.75 to 6.18) | 0.154 | - | - |
| BDI ≥ 14 | 1.82 (0.44 to 7.57) | 0.413 | - | - |
| Berlin score, high risk | 1.09 (0.40 to 2.95) | 0.868 | - | - |
| Difficulty in sleep induction | 0.07 (0.01 to 0.48) | 0.006 | - | - |
| Difficulty in sleep maintenance | 2.97 (0.28 to 30.92) | 0.364 | 0.30 (0.09 to 0.94) | 0.04 |
| Social network size | 1.18 (0.88 to 1.58) | 0.266 | - | - |
| Feeling intimacy in social network | 1.48 (0.83 to 2.65) | 0.187 | 1.93 (1.59 to 2.35) | <0.001 |
| Sharing leisure time with spouse | 0.64 (0.19 to 2.15) | 0.468 | - | - |
| Discussing concerns with spouse | 0.72 (0.11 to 4.83) | 0.734 | - | - |
| Support from spouse | 3.04 (0.88 to 10.54) | 0.079 | - | - |
| Blame from spouse | 0.66 (0.27 to 1.63) | 0.367 | - | - |
| Having friends (≥1) outside of family | 0.68 (0.25 to 1.84) | 0.445 | - | - |
| Bridging potential, yes | 0.60 (0.22 to 1.66) | 0.325 | - | - |
| Abbreviations: BMI, body mass index; BDI, Beck Depression Inventory; OR, Odds ratio; CI, Confidence Interval | | | | |
